# Visceral larva migrans associated with earthworm and gecko ingestion: a case report

**DOI:** 10.1186/1752-1947-6-210

**Published:** 2012-07-18

**Authors:** Tao Yu, Li-Na Zhao, Miao-Jing Fan, Huan Wu, Qi-Kui Chen

**Affiliations:** 1 Department of Gastroenterology, Sun Yat-sen Memorial Hospital, Sun Yat-sen University, 107 Yan Jiang Xi Road, Guangzhou, 510120, People’s Republic of China; 2 Department of Pathology, Sun Yat-sen Memorial Hospital, Sun Yat-sen University, 107 Yan Jiang Xi Road, Guangzhou, 510120, People’s Republic of China; 3 Department of Ultrasound, Sun Yat-sen Memorial Hospital, Sun Yat-sen University, 107 Yan Jiang Xi Road, Guangzhou, 510120, People’s Republic of China

## Abstract

**Introduction:**

Visceral larva migrans is a syndrome caused by an infection with larval helminths, which may result in partial or general pathological changes in host tissues. Due to the difficulty in finding the causative parasites, the diagnosis of visceral larva migrans is generally based on compatible clinical signs, epidemic history, marked eosinophilia and pathological examination, especially positive serological test results and the disappearance of symptoms after specific treatment.

**Case presentation:**

We report here the case of a 21-year-old Chinese man who, having ingested living earthworms and geckos at a witch’s suggestion, presented with fatigue and wordlessness lasting for one year along with elevated transaminase levels for one month. Clinical examination showed eosinophilia, elevated transaminase levels, nodular lesions in his liver and typical pathological characteristics of hepatic visceral larva migrans. After four courses of anthelmintic therapy, our patient presented with sustaining improvement of clinical manifestations and normalization of laboratory data.

**Conclusions:**

Because of the difficulty in making a definite diagnosis, anthelmintic therapy should be performed in patients with a suspected diagnosis of visceral larva migrans based on their epidemic history and presence of typical manifestations, especially when the serological test results are negative. Furthermore, patients with severe parasite infection may require multiple anthelmintic therapies in order to eliminate the parasites.

## Introduction

Visceral larva migrans (VLM) is a syndrome caused by an infection with larval helminths. They invade aberrant hosts, such as human beings, but instead of developing into adult worms, they migrate through host tissues, resulting in partial or general pathological changes. A cause of zoonosis, the parasites can finish their natural life cycles in free-living form, which is vital for maintaining their infection capability. While VLM has a worldwide distribution, it is most commonly seen in the tropical and developing countries, where the sanitary and hygienic conditions are poor.

The clinical manifestations of VLM are varied, depending on which organs are involved. Common clinical manifestations are marked eosinophilia and hyperglobulinemia, caused by the host immune response to the larval helminths [1]. Due to the difficulty in finding the causative parasites, the diagnosis of VLM is generally based on compatible clinical signs, epidemic history, marked eosinophilia and pathological examination, especially positive serological test results and the disappearance of symptoms after specific treatment [2]. Here, we report a case in which the patient was suspected of VLM associated with more than one kind of parasitic infection, caused by the ingestion of earthworms and geckos.

## Case presentation

A 21-year-old Chinese man presented to our hospital with fatigue and wordlessness lasting for one year along with elevated transaminase levels for one month. One year before presentation, suffering from work and love problems, our patient had begun to complain of fatigue accompanied by wordlessness, low mood, occasional vomiting and anorexia. Subsequently, he had been diagnosed with depression disorder and had received anti-depression therapy in his native hospital several times, but to little effect. He turned to a native witch and ingested living earthworms and geckos for 10 days at her suggestion. One month before his admission to our hospital, a laboratory evaluation revealed that his transaminase level was elevated. He denied fever, jaundice, abdominal pain, melena, changes in bowel habits, chest pain, respiratory symptoms or visual changes. Furthermore, in the previous year he had unintentionally lost 25kg in weight. Nothing notable was found in his medical history or previous drug therapy.

His physical examination demonstrated malnutrition, scythropasmus and low voice. No skin eruption was observed. His heart, lung and abdominal examination showed no sign of any abnormality. A neurologic examination did not reveal any focal deficits.

**Figure 1:**
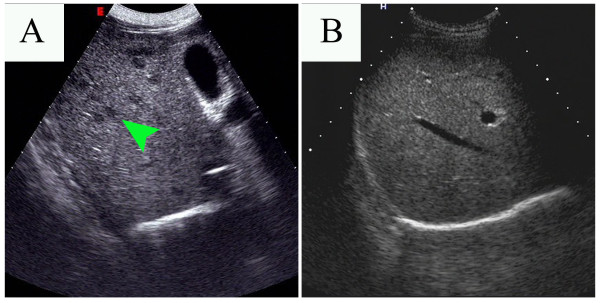
**Abdominal ultrasound results before and after the first therapy. (A)** Before therapy, multiple small hypoechoic nodular lesions were detected (arrow) in the patient’s liver. **(B)** The result of the ultrasound after the first anthelmintic therapy was normal.

**Figure 2:**
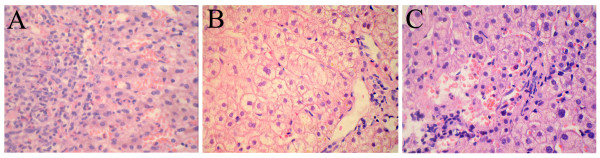
**The evolution of liver pathology throughout the anthelmintic therapies. (A)** Marked edema of hepatocytes, local necrosis, sinusoidal hemorrhage and heavy eosinophil leukocyte infiltration in portal areas were observed before therapy. **(B)** The repeated liver biopsy showed spot necrosis and partial cholestasis with some neutrophil and eosinophil leucocyte infiltration in the portal space after the first therapy. **(C)** The third liver biopsy showed hepatocyte edema accompanied by mild neutrophil and eosinophil leukocyte infiltration in the portal space. Hematoxylin and eosin stain × 400.

Laboratory examination showed a white blood cell count (WBC) of 16.5 × 10^9^/L, with 75.6% eosinophils, alanine transaminase (ALT) 143U/L, aspartate transaminase (AST) 112U/L, gamma-glutamyltransferase (GGT) 176U/L, total bilirubin (TB) 33.1μmol/L, direct bilirubin (DB) 19.0μmol/L and indirect bilirubin (IB) 14.1μmol/L. Multiple stool examinations detected *Trichuris trichiura* ova but no other parasite ova or larvae. A positive reaction in his serum to common parasite antigens was only detected against *Paragonimus*. An abdominal ultrasound (US) detected multiple small and hypoechoic nodular lesions (Figure 1A). A bone marrow biopsy revealed obvious eosinophilia. An US-guided liver biopsy was performed and revealed marked edema of hepatocytes, local necrosis, sinusoidal hemorrhage and heavy eosinophil leukocyte infiltration of portal areas (Figure 2A). No microorganisms were observed.

According to these findings, parasitic infection accompanied by VLM was suspected, combined with depression disorder. A course of albendazole was administrated at a dosage of 400mg once daily for 14 days. One month later, a laboratory examination was conducted and showed WBC 10.4 × 10^9^/L, with 18.14% eosinophils, ALT 88U/L, AST 65U/L, GGT 95U/L, TB 31.2μmol/L, DB 16.0μmol/L and IB 15.2μmol/L. Abdominal US revealed the disappearance of the previous multiple hypoechoic nodular lesions (Figure 1B). A repeated liver biopsy showed spot necrosis and partial cholestasis with some neutrophil and eosinophil leucocytes infiltration in the portal space (Figure 2B).

A second course of anthelmintic treatment (albendazole, 400mg/day) was given for another 14 days. Six months after the second course, a laboratory evaluation showed: WBC 9.3 × 10^9^/L with 10.6% eosinophils, ALT 45U/L, AST 42U/L, GGT 68U/L, TB 28.5μmol/L, DB 13.0μmol/L and IB 15.5μmol/L. No parasite ova or larvae were detected in a repeated stool examination. A repeated abdominal US and bone marrow biopsy were normal. A third liver biopsy showed hepatocyte edema accompanied by mild neutrophil and eosinophil leukocyte infiltration in the portal space, but no necrosis was observed (Figure 2C).

A third course of anthelmintic therapy (albendazole, 400mg/day) was administrated for an additional seven days. Two weeks later, praziquantel was prescribed at a dosage of 1g twice daily for three days (120mg/kg, the fourth course). Two weeks after the fourth course, the laboratory examination results were almost normal except for slightly elevated eosinophil percentage (6.6%). Three months later, our patient presented with increased physical activities and his laboratory data all returned to a normal range (WBC 6.5 × 10^9^/L with 4.0% eosinophils, ALT 27U/L and AST 25U/L).

## Discussion

VLM was first described by Beaver *et al*. in a case series of some children who presented with fever, hepatomegaly, pulmonary infiltration and peripheral eosinophilia as a result of *Toxocara canis* infection [3]. Subsequently, several kinds of parasites, such as *T. catiAscaris suumCapillaria hepaticaGnathostoma spinigerumSpirometra mansoniAngiostrongylus cantonensisPagumogonimus skrjabini* and some *Ancylostoma* species, have been reported to cause VLM [4,5]. Because humans are unsuitable hosts for larval helminths, the parasites, once entering, migrate without growing among the various organs, such as the liver, lungs, brain and eyes, leading to tissue damage and inflammation with eosinophilic infiltration. Among the visceral organs involved, the liver is most commonly affected, due to the portal venous drainage of visceral organs [6]. When larvae penetrate the wall of the gut, gaining access to the portal venous circulation, they move through the liver and induce liver parenchyma necrosis, interstitial edema, hemorrhage and eosinophilic exudates. In addition, their released antigens can stimulate the host immune system, resulting in fever, marked eosinophilia and hyperglobulinemia [1].

Clinical manifestations of VLM involving the liver include fever, fatigue, abdominal pain and hepatomegaly [7]. Laboratory evaluation of patients with VLM almost always reveals leukocytosis with notable eosinophilia and elevated transaminase levels [8]. Sonographic findings of hepatic VLM are multiple ill-defined oval or elongated small nodular lesions scattered in the liver parenchyma [9]. A typical liver biopsy shows central necrosis surrounded by a mixed inflammatory infiltrate with numerous eosinophils and variable numbers of neutrophils, lymphocytes and Charcot-Leyden crystals, which is strongly suggestive of VLM [10]. In this case, our patient had partial clinical manifestations of VLM involving the liver, including fatigue, eosinophilia, elevated transaminase levels, hepatic nodular lesions and typical pathological characteristics. These manifestations led to a diagnosis of hepatic VLM.

However, the diagnosis of VLM should not be just based on typical clinical manifestations. The definite diagnosis of parasite infection should be based on detected worms or eggs, but the causative parasites of VLM are difficult to find. Larval helminths in aberrant hosts are unable to fully mature and reproduce, which makes the stool examination for ova invalid. Furthermore, due to their tiny size and rapid movement in tissues, the larvae are rarely observed in pathologic examination [10]. Therefore, positive serological test results are generally accepted as an indirect method to identify the causative parasite. In this case, a positive serum reaction against *Paragonimus* antigen suggested *Paragonimus* infection. Our patient was from Hainan province in China, where the soil-transmitted parasite is endemic. He also had a history of multiple ingestion of live earthworms and geckos, which can serve as paratenic carriers of soil-transmitted larval helminths such as *Toxocara* larvae and which can carry contaminated soil. Given these factors, soil-transmitted larval helminth infection should be taken into consideration regardless of the negative serological test results [11]. Similarly, in 2006 Cianferoni *et al*. reported the case of a patient with VLM involving the lung and liver through *Toxocara* infection resulting from earthworm ingestion [12].

In clinical situations, VLM is frequently suspected to be caused by more than one kind of parasite infection. Furthermore, soil-transmitted zoonosis is primarily caused by *Nemata* larval infection and more commonly seen involving hepatic damage than *Paragonimus*. Because albendazole is an effective and relatively safe drug against *Nemata* larvae, the first course of anthelmintic therapy administrated in this case was high-dose albendazole [13]. A good clinical response to albendazole treatment was obtained, indicating a correct diagnosis of VLM. After four courses of high-dose anthelmintic therapy (albendazole and praziquantel), all clinical symptoms were improved. The notable eosinophilia gradually disappeared and the elevated transaminase level steadily returned to a normal range. Moreover, the normalization of his bone marrow and liver, shown by examination of the repeated biopsy specimens, also provided evidence for the positive effect of anthelmintic therapy. These favorable clinical responses further confirmed the diagnosis of VLM.

## Conclusions

We present the case of a patient suspected of having VLM involving his liver, resulting from earthworm and gecko ingestion. Our patient’s positive clinical response to multiple anthelmintic therapies with albendazole and praziquantel confirmed the diagnosis of VLM. Due to the difficulty in making a definite diagnosis, anthelmintic therapy should be administered to patients suspected of having VLM according to their epidemic history and presence of typical manifestations, including when serological tests are negative. Furthermore, patients with VLM with severe parasite infection may require multiple anthelmintic therapies to eliminate the parasites.

## Consent

Written informed consent was obtained from the patient for publication of this case report and accompanying images. A copy of the written consent is available for review by the Editor-in-Chief of this journal.

## Competing interests

The authors declare that they do not have any competing interests*.*

## Authors’ contributions

TY and LNZ wrote the paper; MJF confirmed the pathologic diagnosis; HW performed the abdominal ultrasound test; QKC and TY interpreted the patient data regarding VLM and cured the patient. All authors read and approved the final manuscript.
